# Monte Carlo simulations of spherocylinders interacting with site-dependent square-well potentials

**DOI:** 10.1038/s41598-024-53182-1

**Published:** 2024-02-14

**Authors:** Kiranmai Yellam, Anshuman Priyadarshi, Prateek K. Jha

**Affiliations:** Department of Chemical Engineering, IIT Roorkee, Roorkee, Uttarakhand 247667 India

**Keywords:** Thermodynamics, Polymers, Self-assembly

## Abstract

Monte Carlo simulations are performed to study the self-assembly of a dilute system of spherocylinders interacting with square-well potential. The interactions are defined between randomly placed sites on the axis of the spherocylinder, akin to the interacting groups on a rigid rodlike molecule. This model therefore also serves as a minimal coarse-grained representation of a system of low molecular weight or stiff polymers with contour lengths significantly lower than the persistence length, interacting predominantly with short-range interactions (e.g., hydrogen bonding). The spherocylinder concentration, square-well interaction strength and range, and fraction of interacting sites are varied to study the phase behavior of the system. We observe the formation of dispersed, bundled, and network configurations of the system that may be compared with previous atomistic simulation results of weak polyelectrolytes.

## Introduction

The ability of molecules to self-assemble^1^ due to physical interactions between some of their chemical groups has attracted tremendous scientific interest in last few decades and is central to the bottom-up design of novel systems and devices for a range of applications^2,3^. As one particular example, polymers often self-assemble to form dense, matrix structures that can be used to entrap other small molecule drugs and facilitate their controlled release^4,5^. Since the underlying physical interactions that drive this process can be tuned by an intrinsic or extrinsic stimulus (e.g., pH, temperature, light, magnetic field, etc.), the resulting changes in the self-assembly behaviour may also be harnessed to target drug release in specific regions of the human body^6,7^. Previous molecular simulation studies^8,9^ in our group have focussed on the use of pH-responsive self-assembly of weak polyelectrolytes in the controlled/targeted drug release of neutral or ionic drugs.

**Figure 1:**
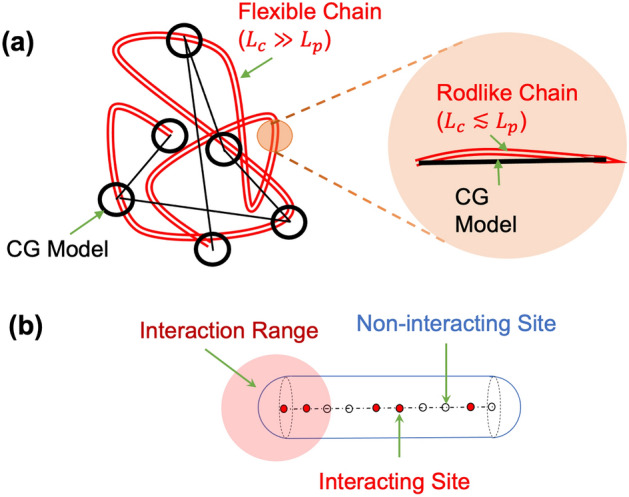
(**a**) Typical polymer chain configuration and appropriate coarse-grained CG models for flexible and rodlike regimes. (**b**) Spherocylinder with interacting and noninteracting sites are considered in this work, which may serve as a CG model for rodlike regime.

Polymer self-assembly can be simulated using both atomistic and coarse-grained polymer models^10–12^. The atomistic models, though more accurate, are limited to low molecular weight polymers due to their huge computational cost. In most cases, the ‘flexible’ nature of polymers is not captured in these simulations^13^, since a polymer chain is essentially ‘rodlike’ if its contour length ${L}_{c}$ is below its persistence lengths ${L}_{p}$ (Fig. 1a). Coarse-grained (CG) simulations (e.g., those using the bead-spring models) do not suffer from this limitation but do not capture atomistic details. If one actually wishes to use CG simulations for low molecular weight polymers where the chain behavior is ‘rodlike’, a polymer chain can also be modeled as a rod or a spherocylinder. Simulations of rods/spherocylinders can also be useful in the study of polymers of high persistence length that remain stiff for much larger molecular weights. Further, when properly trained using atomistic simulation results, these models can also be useful in developing systematic CG models that provides certain insights at par with atomistic simulations, but with much lower computational cost. Such models may be useful, for instance, in studies of self-assembly processes at longer time scales than that typically possible using atomistic simulations.

The transition from an atomistic universe to a coarse-grained universe requires a gross simplification of the molecular representation where several atoms are often lumped into CG units, and the energy of the system may be approximated in terms of the pair potential between the CG units. A naïve strategy to attain this may be the following. One begins by lumping some of the nearby atoms (usually bonded to each other) and determines an “effective” pair interaction energy of the lumped units as the averaged interaction energy of the atoms of these units that are being lumped. As an example, let us consider two CG units ${\varvec{A}}$ and ${\varvec{B}}$ formed by set of atoms $\left\{ {a_{i} } \right\}$ and $\left\{ {b_{j} } \right\}$, respectively. Then, the effective interaction energy between the lumped units may be defined for this naïve strategy as

$$u_{{{\varvec{AB}}}} = \mathop \sum \limits_{i} \mathop \sum \limits_{j} u_{{a_{i} b_{j} }}$$

where $u_{{a_{i} b_{j} }}$ is the pair interaction between a pair of atoms $a_{i}$ and $b_{j}$ of the two units. Though this naïve strategy might appear quite logical, we neglect the effect of pair interaction between the atoms of the same CG unit. Also, there is a considerable difference in the length and time scales pertinent to atoms constituting the CG units and the CG units themselves. Moreover, to perform CG simulations, we need to obtain functional form of $u_{{{\varvec{AB}}}}$ in terms of the distance between the CG units $r_{{{\varvec{AB}}}}$. In practice, one usually begins with simple functional forms of $u_{{{\varvec{AB}}}} \left( {r_{{{\varvec{AB}}}} } \right)$ and obtain the relevant parameters by fitting results of certain target property(s) computed from atomistic simulations and/or observed in experiments. For example, in studies using the iterative Boltzmann method^14^, the radial distribution functions obtained by the CG simulation may be fitted to that obtained by the atomistic simulation by iterative variations of the well depth of the Lennard–Jones (LJ) potential. Since the atomistic simulations of polymers are limited to low molecular weights as discussed earlier, it is sometimes appropriate to also include additional parameters (e.g., radius of gyration)^15^ that are used to fit experimentally obtained behavior for high molecular weights. Before proceeding further, it is important to note that the number of atoms lumped into a CG unit determines the resolution and computational efficiency of the CG model. An increase in the number of atoms per CG unit results in lower resolution but higher computational efficiency. Simulations of polymers often require aggressive CG strategies, where a repeat unit or several repeat units define a CG unit.

In this study, we attempt to develop a minimal CG model of pH-responsive polyelectrolytes, e.g., polyacrylic acid (PAA), of low molecular weights. In a previous atomistic simulation study in our group^16^, simulations were conducted for different PAA concentrations in water and different fraction of deprotonation defined as

$$f_{ - } = \frac{{\left[ {{\text{COO}}^{ - } } \right]}}{{\left[ {{\text{COO}}^{ - } } \right] + \left[ {{\text{COOH}}} \right]}}$$

where $\left[ {{\text{COO}}^{ - } } \right]$ and $\left[ {{\text{COOH}}} \right]$ are the number of deprotonated and protonated carboxylic acid group on PAA. Simulations were also conducted for different patterns of deprotonation (i.e., random and end deprotonation). The extent of hydrogen-bonding interactions between ${\text{COOH}}$ groups drive the formation of ‘aggregate’ and ‘network’ structures for low and intermediate values of $f_{ - }$ and ‘dispersed’ phase is observed due to electrostatic repulsion between ${\text{COO}}^{ - }$ groups for high $f_{ - }$. We have also observed that the short-ranged part of Coulomb interactions and the Lennard Jones interactions are most important driving force for the phase behavior^9^. With this understanding, we hypothesize that only a short-range interaction potential with variable interaction depths in a CG simulation may be sufficient to reproduce similar phase behavior as atomistic simulations. The use of only short-range interaction in a CG simulation is also convenient from computational perspective, as the long-range interactions are much more difficult to handle and would result in higher simulation times.

In this work, we represent the polymer as a spherocylinder with sites representing the repeat units. Sites are further classified as ‘noninteracting’ and ‘interacting’ (Fig. 1b), analogous to repeat units containing ${\text{COO}}^{ - }$ and ${\text{COOH}}$ in PAA, respectively. That is, we can relate the fraction of interacting sites with the fraction of deprotonation as $f = 1 - f_{ - }$. Noninteracting sites have only hard-core interaction (i.e., overlaps not allowed), whereas the interacting sites interact with a short-range interaction represented using a square-well potential. The use of a spherocylinder in place of a cylinder is convenient to implement the hard-core interaction and to minimize possible differences in the behavior near edge sites (at the ends of the cylinder) and face sites (along the length of the cylinder except the ends). More details about the simulation methodology will be discussed in the next section. It is worth pointing out that while this serves as a minimal CG representation of the system under consideration, several intricacies are neglected here that may be considered in future studies. First, the atomistic simulations^16^ also observed a significant hydrogen-bonding between ${\text{COO}}^{ - }$ and ${\text{COOH}}$ groups. Second, we neglect the long-range electrostatic repulsion between ${\text{COO}}^{ - }$ groups and the effect of counterions and solvent. Third, since a repeat unit is considered as a CG unit, the effects of chain tacticity are not considered. Despite these obvious shortcomings, the phase behavior obtained in the atomistic molecular dynamics simulations and Monte Carlo simulations of the CG model is qualitatively similar and the parameters of the CG simulations may, in principle, be fitted to reproduce the atomistic simulation results.

Self-assembly of spherocylinders has been explored in several previous studies. Frenkel and co-authors^17,18^ have obtained the phase diagram of a system of hard spherocylinders with variations in the concentration and aspect ratio. They report a rich phase diagram comprising of isotropic, liquid crystalline (nematic/smectic), and crystalline phases. Phase diagram of spherocylinders with a generalized square-well attraction has also been studied in their subsequent study^19^, where the objective was to model depletion attractions induced in spherocylinder colloids by nonadsorbing polymer particles. The formation of 2D crystalline films of spherocylinders has also been recently reported in an experimental and computational study^20^. Self-assembly of mixtures of spherocylinders with spheres has also been studied^21^. Unlike the above mentioned studies, we are interested in a dilute solution of spherocylinders, with variable interaction strengths along its length. We study the effects of variations of spherocylinder concentration, interaction strength, and fraction of interacting sites. Study of polymer phase behavior is important for its potential use in various applications. For example, the reversible self-assembly of polymer may be useful in the development of stimuli-responsive polymeric carriers for drug delivery^6,22^.

## Methodology

Spherocylinder is modeled as a cylinder with hemispherical caps at the two ends. The diameters of the cylinder and hemispherical caps are identical and is used as the length scale ($\sigma$). We work with dimensionless variables in this work (Table 1), using $\sigma$ as the length scale and ${k}_{B}T$ as the energy scale, where ${k}_{B}$ and $T$ are the Boltzmann constant and absolute temperature, respectively. The length scale may be taken as the size of a repeat unit for comparison with atomistic simulations. In order to simulate similar concentrations as ref.^16^, we keep the simulation box size as $L=24$ (dimensionless) that is equivalent to the box size of 6 nm in ref.^16^, using $\sigma =0.25 nm$. Similarly, the number of sites per spherocylinder (${n}_{g}=20$) is taken the same as the number of repeat units in the atomistic simulation work. Using these values, the site concentrations for $N=\mathrm{4,8},\mathrm{16,24}$ are same as the concentration values (0.615 M, 1.223 M, 2.456 M, 3.689 M) simulated in ref.^16^. Henceforth, all the variables are mentioned in a dimensionless form unless otherwise specified. Cases corresponding to different concentration values are referred by the value of $N$, which may be compared with the number of polymer chains in ref.^16^. It should be noted that the overall purpose of the work is to develop a minimal CG model corresponding to previous atomistic simulations^16^. In practice, one needs to perform such simulations in an iterative fashion^14^ to find the correct CG parameters that fit atomistic simulation results. Even though we have not performed such fitting, we have kept the system size similar to that system to facilitate comparison with atomistic simulation results. It is also worth mentioning that the interactions are defined not between the $N$ spherocylinders but between the $N{n}_{g}=\mathrm{80,160,320,480}$ sites of spherocylinders, thus providing reasonable statistical significance to our simulations. Further, in order to test the validity of our predictions, we have also performed some comparisons with larger system sizes, which will be discussed in the Results and Discussion section.

**Table 1:** Dimensionless variables used in this study.

Description	Notation	Value(s)
Length of simulation box	$L$	24
Number of spherocylinders	$N$	4, 8, 16, 24
Number of sites	${n}_{g}$	20
Length of spherocylinders	$l$	20
Threshold range of square-well potential	${r}_{0}$	1.2, 1.4, 1.6
Depth of square-well potential	$\varepsilon$	0.2, 0.4, 0.6, 0.8, 1.0, 1.2, 1.5, 2.0
Fraction of interacting sites	$f$	0.2, 0.4, 0.6, 0.8, 1.0

**Figure 2:**
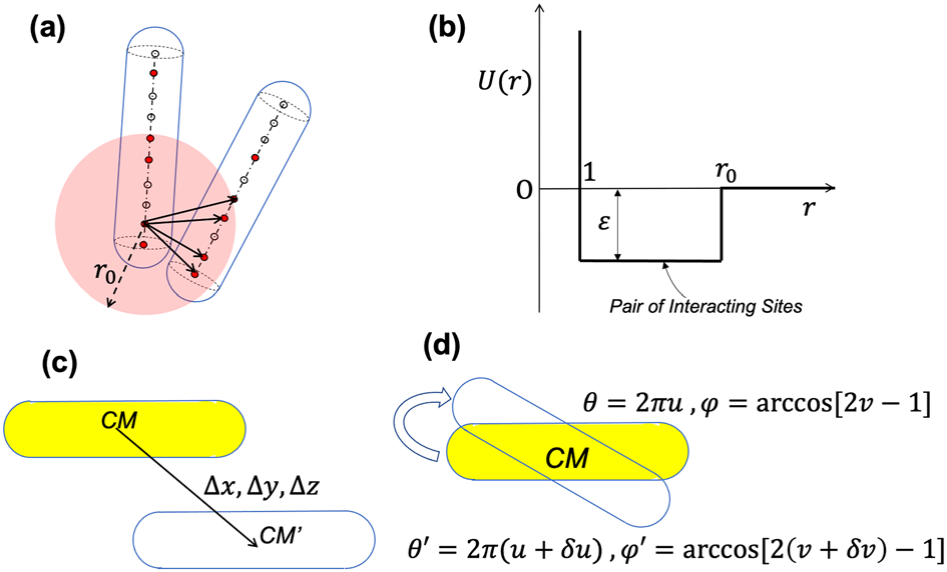
(**a**) Example of a system of interacting spherocylinders showing the typical interaction between sites. (**b**) Interaction energy versus distance for a pair of interacting and interacting sites on spherocylinders. (**c**) The translation move defined by a random displacement $\Delta x,\Delta y,\Delta z$ in a direction perpendicular to the axis. (**d**) The rotation move defined by a change in azimuthal angle $\theta$ and polar angle $\varphi$ to $\theta^{\prime }$ and $\varphi^{\prime }$ respectively. See text for description.

Simulations are performed for a system of $N$ spherocylinders of length $l$ in a cubic simulation box of length $L$. ${n}_{g}$ sites are uniformly placed along the axis of the spherocylinder including its ends. A fraction $f$ of these sites are randomly chosen as ‘interacting’, which interact with other ‘interacting’ sites via a square-well potential of depth $\varepsilon$. Figure 2a shows the square-well interaction pairs for two spherocylinders and Fig. 2b shows the square-well potential. The hard core interaction between spherocylinders is implemented by computing the minimum distance $r_{m}$ between spherocylinder axes defined as line segments as in Fig. 2a ^23^ and ensuring that $r_{m} \le 1$. Our choice of square-well potential draws some similarity with ‘hydrogen bonds’ defined as geometric criteria in atomistic simulations^16^, where the hydrogen bonds are assumed to be formed if a donor–acceptor pair is within a threshold distance ($\sim 0.35$ nm) and hydrogen-donor–acceptor angle less than $30^\circ$. In our case, $r_{0}$ may be considered as the threshold distance and the interacting sites may be considered as donor/acceptor. However, no angle criterion is included here, since the hydrogen atoms are not explicitly modeled. In this study, we loosely refer to the pair of sites of different spherocylinders within a distance $r_{0}$ as ‘hydrogen bonds’. However, in order to study the possibility of other types of short-range interactions, we vary $r_{0}$ and $\varepsilon$ to obtain a phase behavior of the system. Therefore, statistics of ‘hydrogen bonds’ reported in this study are not referring to actual hydrogen bonds but a close contact between sites that may also be formed by any other type of interaction (e.g., ionic complexation). Note that our choice of square-well potential is only made to develop a minimal CG model with fewer parameters. Of course, the use of Lennard–Jones (LJ) potential or Morse potential in place of square-well potential could have been more justified for a general treatment, but will introduce additional model parameters. In the case of LJ and Morse potentials, the attractive part of the interactions smoothly decreases with increasing distance, whereas it is constant in the attraction region for the square-well potential (Fig. 2b).

Monte Carlo (MC) simulations are performed in canonical (NVT) ensemble with periodic boundary conditions. Random distribution of spherocylinders in the simulation box is taken as the initial configuration of the system. MC Simulations are performed for $2 \times 10^{8}$ sweeps, where a sweep consists of $N$ MC steps. In each MC step, a spherocylinder is randomly chosen and a trial translation/rotation is attempted. Both translational and rotational moves are performed with 50% probability for each of the moves, and the moves are accepted/rejected using the Metropolis criteria. Translation moves (Fig. 2c) involve a random displacement ${\Delta }x,{\Delta }y,{\Delta }z \in \left( { - 0.05, 0.05} \right)$ of the spherocylinder, in a direction perpendicular to its axis. The rotation around the center of the mass of the spherocylinder (Fig. 2d) is defined in terms of $\theta \in \left[ {0,2\pi } \right]$ and $\varphi \in \left[ {0,\pi } \right]$, which in turn are defined in terms of variables $u,v \in \left[ {0,1} \right]$ such that $\theta = 2\pi u$ and $\varphi = {\text{acos}}\left( {2v - 1} \right)$. In a rotation move, we increment $u$ and $v$ by $\delta u \in \left[ { - 0.05,0.05} \right]$ and $\delta v \in \left[ { - 0.05,0.05} \right]$. It is to be noted that the choice of translation moves as only being perpendicular to the axis and the rotation moves about the centre of mass has only been made for simplicity considerations in this study and other moves satisfying detailed balance criteria may also be considered. Simulations are performed using an inhouse serial code on an Intel Xeon workstation with 3.5 GHz turbo processor. The time needed for the simulation varied with the system size up to ~ 7 days for $N = 24$ system.

MC simulations involve an ‘equilibration phase’, which is monitored by tracking the total number of hydrogen bonds, $H$ (i.e., number of pairs of sites with distance within $r_{0}$). Since the hydrogen bonding is the only interaction (apart from hard-core) in this work, we could have equivalently tracked the energy. However, in our previous atomistic simulation studies on polymer aggregation^24^, we observed that the number of contacts/hydrogen bonds is a somewhat better measure for aggregation behavior. Equilibration phase is followed by a ‘production phase’, which is used for computation of average number of hydrogen bonds per site $\left\langle h \right\rangle$ obtained by dividing the average value of $H$ by the total number of sites in the system $Nn_{g}$. Note that although it may seem more appropriate to consider only interacting sites for normalization, we consider all sites in order to compare with the aggregation behavior of spherocylinder even in cases when the number of interacting sites is $\approx 0$. In practice, a long simulation run is conducted and the first ~ 70% of the simulation run is discarded, considering the need for equilibration. The average properties are computed for last ~ 30% of simulation run, defined as the production phase. The average number of hydrogen bonds per site $\left\langle h \right\rangle$ has been calculated for every simulation as 2 times the total number of hydrogen bonds divided by the number of sites. Here, a factor of 2 comes as 2 sites participate in the formation of a hydrogen bond. In order to access the extent of the ordering of the spherocylinders, we make use of the liquid crystalline order parameter^25^,

$$\left\langle S \right\rangle = \frac{1}{2}\left\langle {3\cos ^{2} \theta - 1} \right\rangle$$

which has a maximum value of $1$ when the spherocylinders are perfectly aligned within a bundle. $\left\langle S \right\rangle$ is also tracked along with $H$ during the simulation run and is averaged over the MC steps for the production run.

## Results and discussion

**Figure 3:**
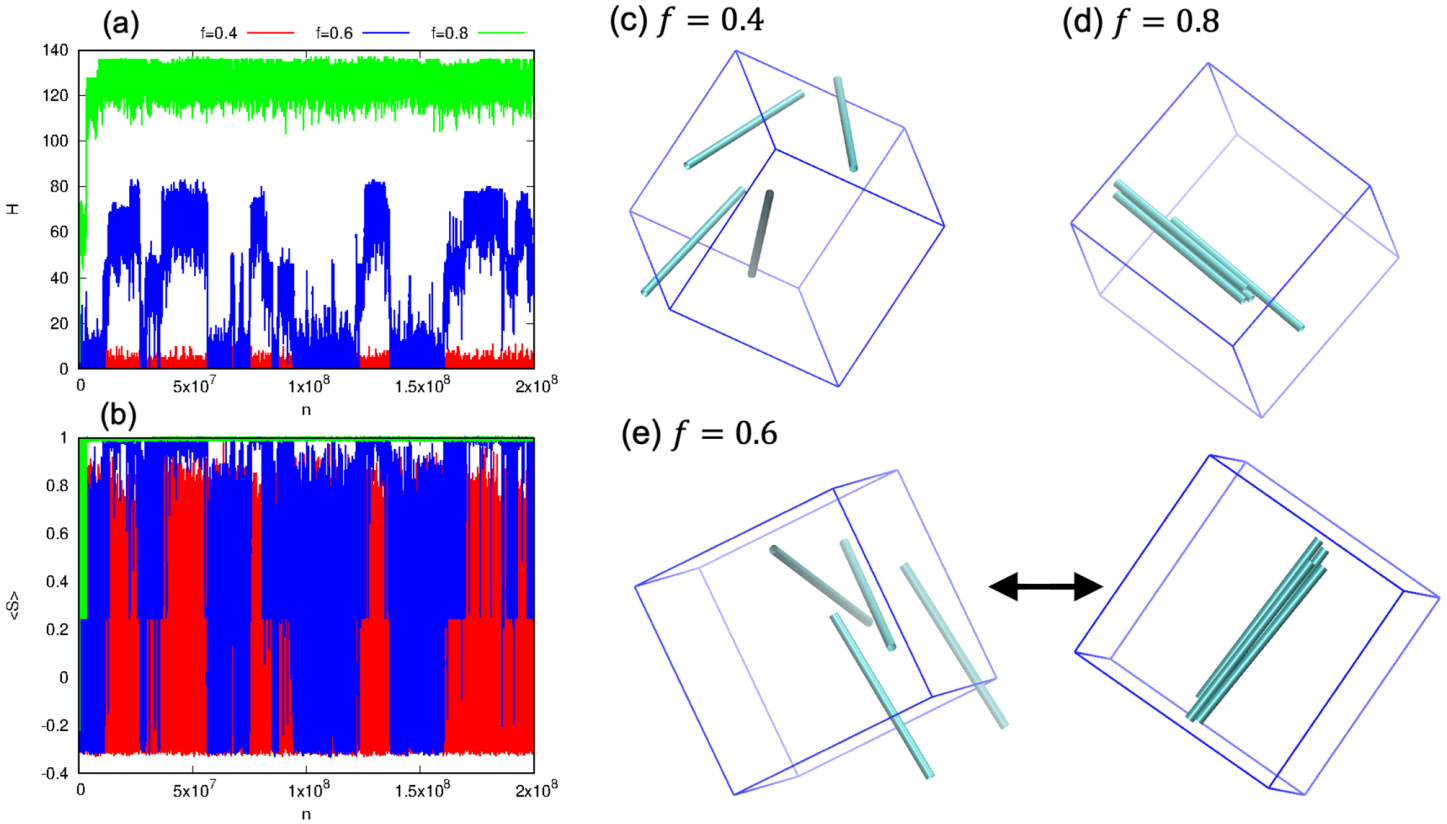
(**a**, **b**) Shows the number of hydrogen bonds and average order parameter, respectively, against the number of MC steps for three different fraction of interacting sites ($f$). Color legends for $f$ given on top are the same for both (**a**, **b**). (**c**, **d**) shows the dispersed and bundled phase formed for $f = 0.4$ and $f = 0.8$, respectively. For $f = 0.6$ case, a reversible transition between the dispersed and bundled phase is obtained as shown in (**e**). Other model parameters are $N = 4$ , $r_{0} = 1.4$ and $\varepsilon = 0.8$. The caps of spherocylinders are not shown.

Figure 3 shows the simulation results for three different $f$ values for $N = 4$ case with $r_{0} = 1.4$ and $\varepsilon = 0.8$. For $f = 0.4$, we observe a dispersed phase (typical snapshot in Fig. 3c) that is characterized by small values of both $\left\langle h \right\rangle \approx 0.13$ and $\left\langle S \right\rangle \approx 0.02$. For $f = 0.8$, on the other hand, a bundled phase is obtained with much larger values of $\left\langle h \right\rangle \approx 130$ and $\left\langle S \right\rangle \approx 1$ (perfect ordering). Interestingly, for $f = 0.6$, we observe a reversible transition between dispersed and bundled phases, characterized by large oscillations in $\left\langle h \right\rangle$ and $\left\langle S \right\rangle$ values (blue line in Fig. 3a,3b and snapshots in Fig. 3e). That is, while the hydrogen bonds form when the spherocylinders come near to each other, they are relatively fragile (compared to Fig. 3d) and break to form a dispersed phase again. In other words, the lifetime of these hydrogen bonds are quite small. Similar oscillations have been reported in a previous study of janus particle self-assembly^26^. Overall, our results can be explained by considering the competition between thermal and interaction energy, corresponding to entropic and enthalpic contributions respectively. At lower values of $f$, the thermal energy dominates resulting in the formation of dispersed phase. At a higher value of $f$, the interaction energy dominates resulting in formation of bundled phase. In between these two extremes, when both the interaction and thermal energy are comparable in magnitude, the reversible transition between the bundled and dispersed phases may be observed.

**Figure 4:**
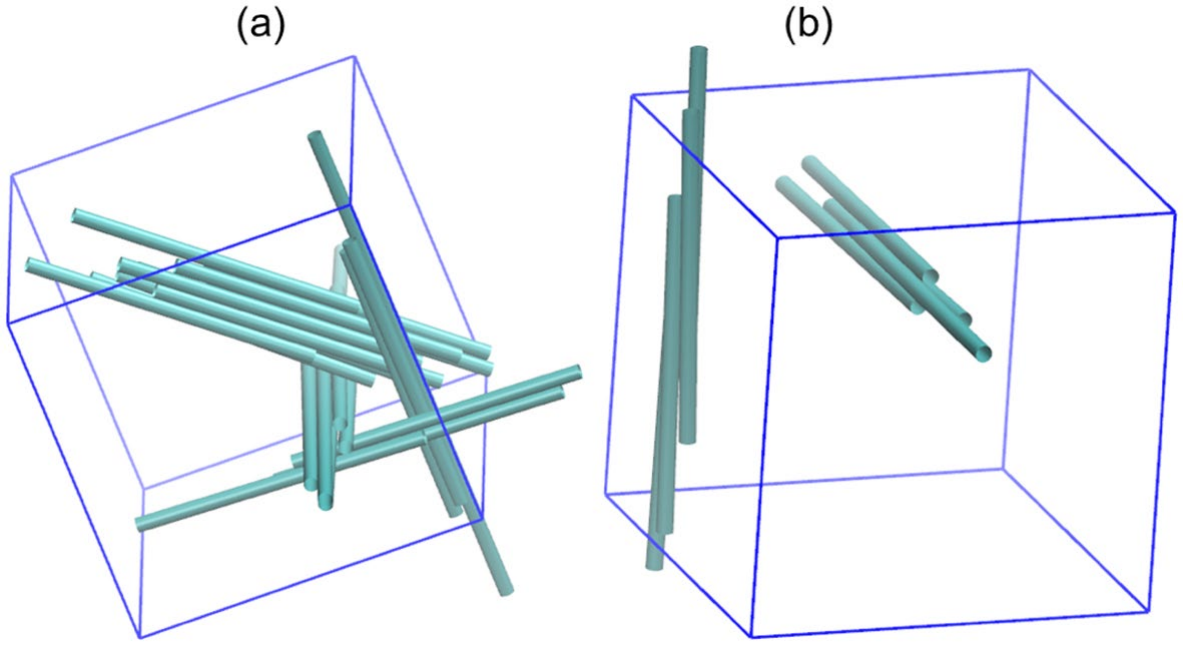
Examples of kinetically trapped structures formed in simulations for some cases: (**a**) Network formed for $N = 24, \varepsilon = 0.2, r_{0} = 1.2, f = 0.6$, and (**b**) Multiple bundles formed for $N = 8, \varepsilon = 0.2, r_{0} = 1.2, f = 0.6$.

We classify the obtained structures into ‘bundled’, ‘network’ and ‘dispersed’ phases. Dispersed phase forms for low interaction strengths, which refers to the absence of aggregation. These may be characterized by $\left\langle h \right\rangle \approx 0$ and $\left\langle S \right\rangle \approx 0$*.* For intermediate interaction strengths, the spherocylinders assemble and align together to form bundles, characterized by larger values of $\left\langle h \right\rangle$ and $\left\langle S \right\rangle \approx 1$. For high interaction strengths, we sometimes observe the formation of an interconnected network structure (Fig. 4a) or multiple bundles (Fig. 4b) that form when the spherocylinders are not able to align themselves due to kinetic limitations. Such kinetic trapping^27^ during self-assembly processes is well known. Network is more likely to form at higher concentrations due to the increased possibility of spherocylinder overlaps during reorientation, while the formation of multiple bundles is more likely at lower concentrations as the formed bundles need to traverse long distances to combine together. Both these cases are characterized by relatively smaller values of $\left\langle h \right\rangle$ (compared to bundled phase) and the $\left\langle S \right\rangle$ value intermediate between 0 and 1 (but not $\approx 0$ or $\approx 1$), and are distinguished by visual observation. Although the use of cluster MC moves^28,29^ may drive them into a bundled phase, we limit them to simpler MC moves and report the kinetically trapped structures as such. This is because such structures are also observed in atomistic MD simulations^16^ due to time scale limitations.

**Figure 5:**
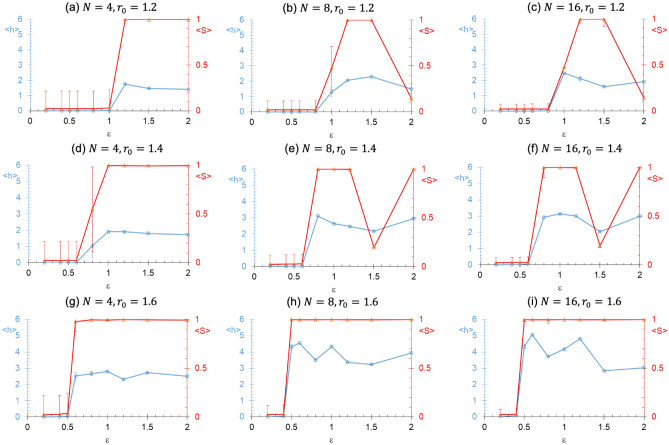
Average number of hydrogen bonds per site $\left\langle h \right\rangle$ and the average order parameter $\left\langle S \right\rangle$ against the interaction depth $\varepsilon$ for different values of the number of spherocylinders $N$ and interaction range $r_{0}$. $f = 0.6$ for all these simulations. $\left\langle h \right\rangle$ and $\left\langle S \right\rangle$ are shown in blue and red color, respectively. The error bars indicate the standard deviation.

Figure 5 shows the variation of $\left\langle h \right\rangle$ and $\left\langle S \right\rangle$ with the interaction depth $\varepsilon$ for different values of $N$ (left to right) and $r_{0}$ (top to bottom). In general, we observe an increase in $\left\langle h \right\rangle$ with an increase in $N$ (or the spherocylinder concentration as the simulation box length is fixed), since an increase in spherocylinder concentration increases the likelihood of contacts. Also, one may expect an increase in $\left\langle h \right\rangle$ with an increase in $\varepsilon$ due to increase in interactions. While this trend is observed for lower values of $\varepsilon$ (${ \lesssim }1$), some deviations are observed for larger $\varepsilon$ values due to kinetic trapping of formed structures. Specifically, the threshold for transition from dispersed phase to bundled phase with an increase in $\varepsilon$ shifts to lower $\varepsilon$ values for higher spherocylinder concentration or higher interaction range. However, $\left\langle h \right\rangle$ does not monotonically increase with a further increase in $\varepsilon$ beyond above mentioned threshold due to the formation of kinetically trapped structures at some of the $\varepsilon$ values, which are characterized by low values of $\left\langle S \right\rangle$. Further, the kinetically trapped structures are less likely to form for higher values of $r_{0}$, as the spherocylinder interacts with a larger number of spherocylinders. It is also important to point out that the formation of kinetically trapped structures might depend on the chosen initial state of the system (Figure S2).

**Figure 6:**
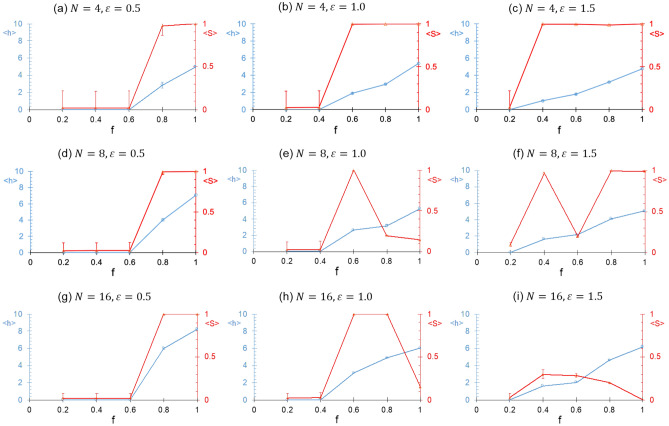
Average number of hydrogen bonds per site ($\left\langle h \right\rangle$) against the fraction of interacting sites ($f$) for different values of number of spherocylinders ($N$) and interaction depth ($\varepsilon$). $r_{0} = 1.4$ for all these simulations. $\left\langle h \right\rangle$ and $\left\langle S \right\rangle$ are shown in blue and red color, respectively. The error bars indicate the standard deviation.

Figure 6 shows the variation of $\left\langle h \right\rangle$ and $\left\langle S \right\rangle$ with the fraction of interacting sites $f$ for different values of $\varepsilon$ (left to right) and $N$ (top to bottom). As expected, the threshold of transition from the dispersed phase to bundled phase with an increase in $f$ shifts to lower values of $f$ with an increase in $\varepsilon$. No change in this threshold is however observed with an increase in $N$, but $\left\langle h \right\rangle$ increased with $N$. Again, network or multiple bundles are observed with further increase in $f$ beyond the above threshold due to kinetic limitations, especially for higher values of $\varepsilon$ and $N$ and lower values of $r_{0}$. We also observe that the order parameter $\left\langle S \right\rangle$ clearly differentiates the ordering within phases. It is worth noting that a decrease in $\left\langle S \right\rangle$ is not always accompanied by a decrease in $\left\langle h \right\rangle$. That is, the extent of ordering characterized by $\left\langle S \right\rangle$ is not directly related to the extent of hydrogen bonding. Thus, both $\left\langle h \right\rangle$ and $\left\langle S \right\rangle$ should be used to interpret the aggregation and ordering characteristics of the spherocylinders.

**Figure 7:**
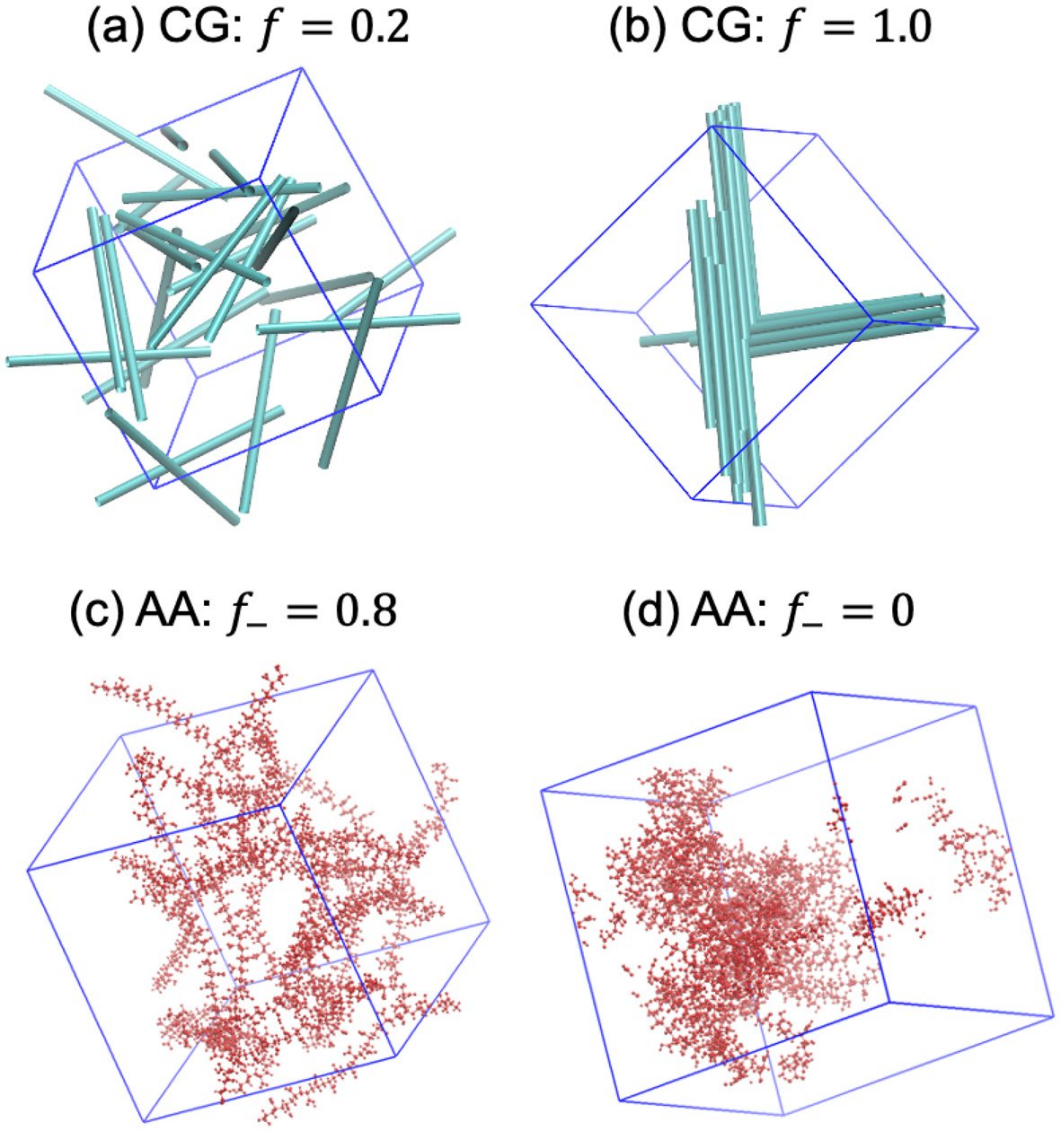
Comparison of CG (this work) and atomistic simulations^16^. (**a**, **b**) shows the typical snapshots of CG simulations for different values of $f$. Other model parameters are $N = 24$, $r_{0} = 1.4$, and $\varepsilon = 1$. Typical simulation snapshots of atomistic simulation for corresponding values of $f_{ - } = 1 - f$ are also reproduced in (**c**, **d**). PAA monomer concentration in atomistic simulation is 3.689 M. The caps of spherocylinders are not shown.

Figure 7 shows a comparison of our CG simulations with the previous atomistic simulation study^16^ for similar concentration in $N = 24$ case and for $f_{ - }$ values of atomistic simulations corresponding to the $f$ values in the CG simulations. The results of the CG simulation study are qualitatively similar to the atomistic simulation results, as dispersed (Fig. 7a and 7c) and bundled phase (Fig. 7b, Fig. 7d) are obtained in both cases. Further, the CG model may be trained by obtaining the value of $\varepsilon$ and $r_{0}$ that provides the same $\left\langle h \right\rangle$ value as atomistic simulation. However, it is worth pointing out that a monotonic trend of $\left\langle h \right\rangle$ with $f_{ - }$ was not observed in atomistic simulations, which was attributed to the existence of hydrogen bonding between ${\text{COO}}^{ - }$ and ${\text{COOH}}$ groups. While we have also observed a nonmonotonic trend in CG simulations due to the formation of kinetically trapped structures, we have considered ${\text{COO}}^{ - }$ groups as noninteracting sites. It should be noted that the formation of kinetically trapped structures (e.g., network) was also observed in atomistic simulations, which therefore cannot be ruled out as the reason for the nonmonotonic trend in that case as well. One other limitation of the current approach is that it ignores the effect of orientation (donor–acceptor-donor angle) in hydrogen bonding. Thus, in order to facilitate a comparison of atomistic and CG simulations, $\left\langle h \right\rangle$ should be similarly defined in the atomistic simulations.

In this work, we have performed our simulation for a statistically small number of spherocylinders or small system size, to facilitate comparison with previous atomistic simulations. Also, the presented statistics are obtained using only one simulation run for each system. Simulations of larger system sizes and averaging over large number of runs (with different random seeds) are naturally expected to yield improved statistics. Figure S1 shows the comparison of $\left\langle h \right\rangle$ obtained in Fig. 5 with that of a larger system with same spherocylinder concentration but twice the simulation box size than the system defined in Table 1, where the averaging is also performed over three independent runs of different random seeds. Although the results do indicate a system-size dependence, the trends are still captured satisfactorily in the smaller-size system presented here. Further, it is important to emphasize that similar issues do always exist in the atomistic simulations of polymeric systems. In this case, once the minimal CG model is trained using atomistic simulation data, CG simulations may be performed for much bigger system sizes to obtain improved statistics. We are planning to pursue this in our future work.

## Conclusion

In this work, we present a minimal coarse-grained model to study the self-assembly of pH-responsive polymers of low molecular weights and/or high persistence length. The polymers are modeled as spherocylinders with sites located along their length, which are characterized as interacting or noninteracting. We study the phase behavior of the system with variations in spherocylinder concentration, interaction strength, and interaction range. We observe the formation of dispersed and bundled phases for low and high interaction strengths, respectively. In some cases of high interaction strength, we observe the formation of kinetically trapped networks or multiple bundles. The phase behavior obtained in the CG simulations shows close resemblance with previous atomistic simulation study. Further, the interaction strength and range may be varied to fit the atomistic simulation results, thus resulting in a systematic CG strategy.

Several possible extensions of this study may be thought of. This model may be used within a systematic coarse-grained approach for systems comprising of stiff or low molecular weight species. One may also consider extending the model to a binary system of spherocylinders representing a mixture of different polymers or polymers with other small molecules (e.g., drugs). Also, the model may be extended to account for somewhat more complex model potentials (e.g., Morse potential or Lennard–Jones potential) in order to extend the applicability of this approach, which will however necessitate the introduction of additional model parameters. The use of cluster moves for efficient equilibration of the kinetically trapped structures may also be considered, which was purposely not considered in this study as such structures also form in atomistic MD simulations. The use of machine learning for the determination of the coarse-grained model parameters^30,31^ using atomistic simulation results could also be explored. Some of these lines of thought will be considered in our future studies.

### Supplementary Information

Supplementary Information.

## Data Availability

The datasets generated during the current study are available from the corresponding author on request.
