# Supplementary material for: Monte Carlo simulations of spherocylinders interacting with site-dependent square-well potentials
Source: Sci Rep. 2024 Feb 14;14:3753. doi: 10.1038/s41598-024-53182-1 (PMC10866863; doi:10.1038/s41598-024-53182-1)
Supplement: Supplementary file 1 — Supplementary Information. [file 41598_2024_53182_MOESM1_ESM.pdf]

Monte Carlo Simulations of Spherocylinders Interacting with Site-Dependent Square-Well Potentials

Kiranmai Yellam, Anshuman Priyadarshi, Prateek K. Jha*

Department of Chemical Engineering, IIT Roorkee, Uttarakhand, India, 247667

*Corresponding author, Email: prateek.jha@ch.iitr.ac.in , Telephone: +91 1332 284810

Supporting Information

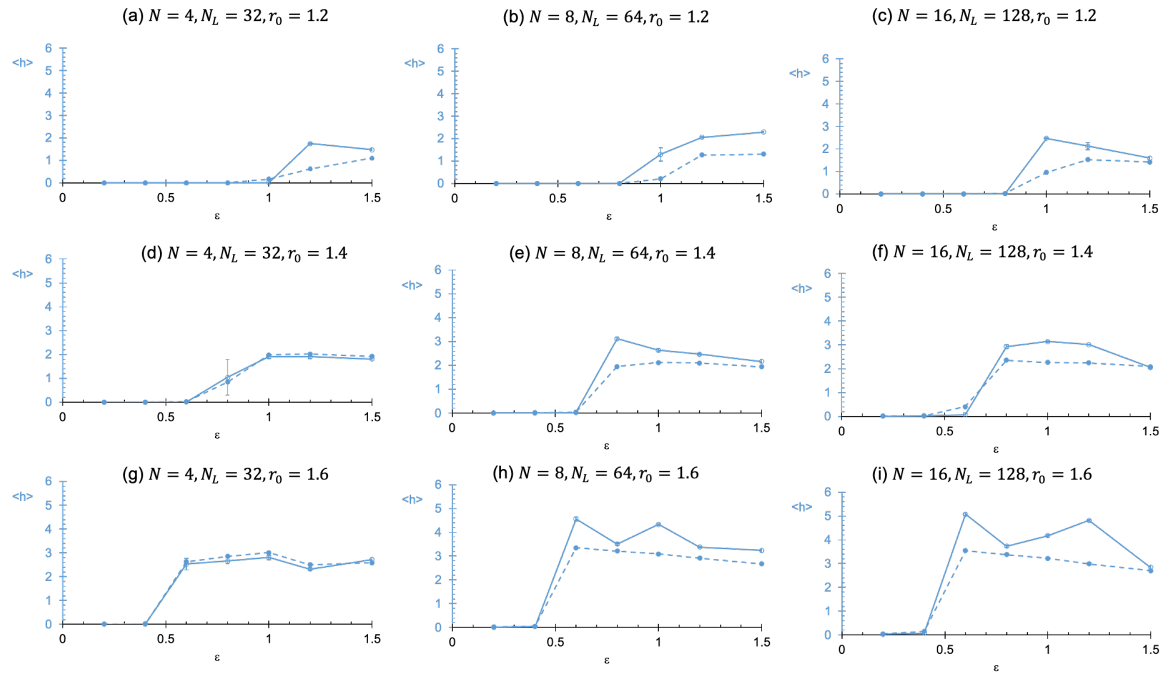

Figure S1: Bold lines show the average number of hydrogen bonds per site $\langle h \rangle$ *and the interaction depth* ε *for different values of the number of spherocylinders* N *and interaction range* r_0 *as given in Figure 5 of the main paper. Dashed lines show the results for systems of same concentration of spherocylinder but with simulation box size twice than that of the bold line, and the results averaged over three runs with different random seed values.* N_L *values indicate the number of spherocylinders for the large system, which is eight times the number of spherocylinders* N *in the system studied in Figure 5.* $f = 0.6$ *for all these simulations. The error bars of bold lines indicate the standard deviation.*

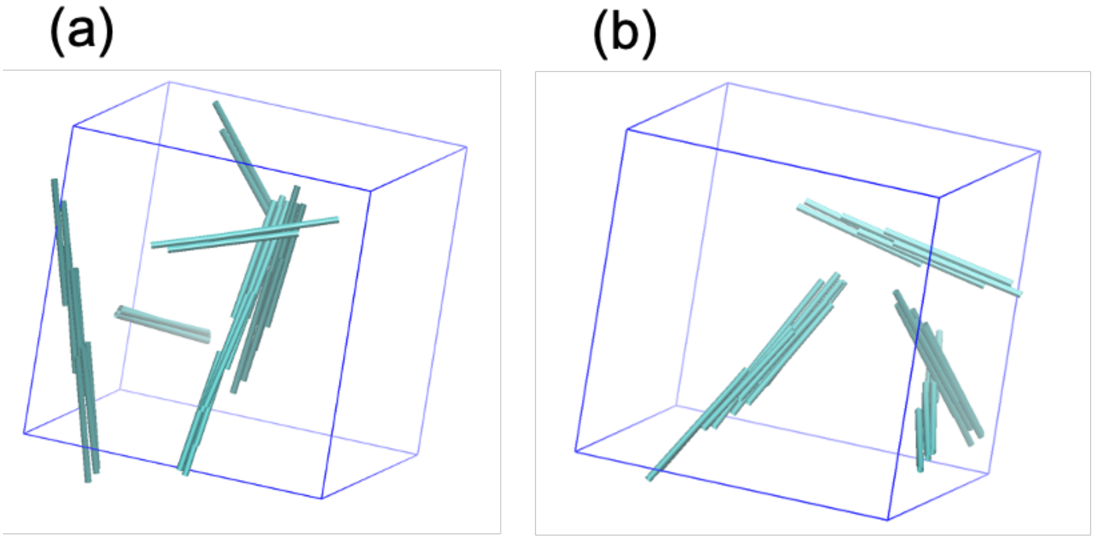

Figure S2: Examples of kinetically trapped structures formed for same model parameters but different initial states. Model parameters: $N = 32$, $L = 48$, $\varepsilon = 1$, $f = 0.6$, $r_0 = 1.4$.
